# Disintegration in the Age of COVID-19: Biological Contamination, Social Danger, and the Search for Solidarity

**DOI:** 10.1177/00027642221132176

**Published:** 2022-10-29

**Authors:** Seth Abrutyn

**Affiliations:** 1 Department of Sociology, University of British Columbia, Vancouver, BC, Canada

**Keywords:** COVID-19, contamination, social solidarity, disintegration, anomie

## Abstract

Like any disaster, COVID-19 laid waste to infrastructure and the ability for a community to *do* community. But, unlike a tornado or nuclear meltdown, COVID-19 laid waste to *social* infrastructure in unique ways that only a disease can do. On the one hand, a pandemic brings biological dangers that, in turn, make all individuals—loved ones, too—into potential threats of biological contamination. On the other hand, the efforts to contain diseases present social dangers, as isolation and distancing threatens mundane and spectacular ritualized encounters and mask-wearing heighten our awareness of the biological risk. By exploring the link between disasters and disease, this paper leverages the contamination process, beginning first with the barriers it presents to making and remaking the self in everyday life. Constraints on ritualized encounters, both in terms of delimiting face-to-face interaction and in determining that some spaces have contaminative risks, reduces collective life to imagined communities or shifts to digitally mediated spaces. The former intensifies the sense of anomie people feel as their social world appears as though it were disintegrating while the latter presents severe neurobiological challenges to reproducing what face-to-face interaction habitually generates. Finally, these micro/meso-level processes are contextualized by considering how institutions, particularly polity but also science, manage collective risk and how their efficacy may either contribute to the erosion of solidarity or provide a sense of support in the face of anomic terror. Using the US to illustrate these processes, we are able to show how an inefficacious State response weakens the already tenuous connective tissue that holds a diffuse and diverse population together, while also exposing and intensifying existing political, economic, and cultural fissures, thereby further eroding existing solidarity and the capacity to rebuild post-pandemic cohesion.

Why was 2020 so traumatic? The answer to this question seems obvious: on the surface, a pandemic rapidly reconfigured the routine going’s on of everyday life, introduced a serious risk to the health of ourselves, our loved ones, and our communities, and, subsequently, ruined the economy and, in the United States at least, triggered an intense summer of social justice activism on behalf of Black people (and, to a lesser extent, all people of color). *Prima facie*, all of this is true. But, sociologically, what is so interesting about a pandemic? The socio-historical study of disease and plague clearly informs us (McNeill, 1976), but what is unique about the 21st century interface between the world today and a plague? How is it similar and, more interestingly, how is it different from the wide array of disasters and exigencies that social scientists study (Arcaya et al., 2020; Erikson, 1994; Vaughan, 1999)? And, finally, why has the pandemic been so exponentially traumatic in the United States, as compared to other similar nations?

Notably, the current pandemic shares many qualities of other massive disruptions, though usually structural and cultural in nature and cause, that served as a significant motivation for the scientific study of society (Nisbet, 1993). In place of the presumed unchanging nature of agrarian life rose an impersonal society governed by formal rules and bureaucratic efficiency (Weber, 1978); a depersonalized society characterized by anonymous, heterogeneous, dense urban spaces (Simmel, 1959); a differentiated society defined by overindividuation, the erosion of social support, and unyielding change in personal and collective values and norms (Durkheim, 1897 [1951]). Though there are many consequences of disruption, the problem of integration remains central to the sociological project—that is, how do collectives generate solidarity and protect against threats to the collective? The following essay foregrounds questions about the pandemic and integration against a Durkheimian backdrop that emphasizes what was perhaps the eponymous theorist’s greatest nightmare: the rapid and potentially fatal disintegration of human societies (Abrutyn, 2019).

Over the course of Durkheim’s career, he proposed two trans-historical mechanisms and one more “recent” mechanism that produce solidarity: (1) micro-level ritualized encounters in which members plunge themselves in the “waters” of the group and renew their commitment and (2) meso-level “communities,” like professional associations, that make group life more tangible than “society”; both of which strengthen the lone macro-level mechanism of cohesion, namely, (3) the state, which uses integrating forces like law, to homogenize diverse populations. These mechanisms serve two purposes: to ensure the reproduction of social life, supplying individuals with meaning and purpose and collectives with motivated actors and second, as protective forces against acute blows to the collective, whether endogenous or exogenous. In the 19th century, acute blows emerged out of economic and political revolutions that radically reconfigured the structure of society, raising questions about how large, diverse, and geographically dispersed societies and their smaller communities could sustain a sense of “we-ness,” if at all. But, these same disruptions have been with human societies forever in the form of disasters of human and natural “design.” The current pandemic offers an opportunity, then, to explore how disease as a disaster threatens these mechanisms of solidarity, thereby contributing to both the disaster scholarship and the sociology of disease.

To do so, I begin by thinking more generally about the sociology of disease as a class of disaster. Like a tornado or other natural disasters (Wallace, 2003), plagues are rapid and destructive, and yet they are different in many ways: invisible in their path and in their infection. Similarly, pandemics share qualities with human-made disasters, like nuclear meltdowns, as plagues are very often the indirect result of human activities like irrigation (McNeill, 1976). Plagues remain more primitive, recurring across time and space; more totalistic having direct and immediate effects on the biological, psychological, and sociological levels; and, the danger of disease is not posed by the environment like climate change, but rather *people*, both intimate and impersonal others. For these reasons, and others, there is temptation to immediately reach for the consequences for solidarity at the macro-level like Durkheim did in, say, *Suicide*. However, there is far more variation in time and space at the macro-level, which serves as a backdrop against the more ubiquitous micro/meso-level experience of anomie in the face of threats to or disintegration of the connective tissue that usually goes unnoticed. Here, in the realm of everyday life, the phenomenological and structural horrors unleashed by the contaminative potential of disease make all types of interactions risky and, in turn, undermine the foundation of meso-level solidarity. Once these generic consequences of disease diffuse across a population, the macro-level space becomes important. The state, as we all saw, must protect its citizens, but no state in history can do or does this equitably and, worse, efficaciously or competently. Moreover, the state, like the micro-/meso-levels of solidarity, is shaped by the historical context in which the pandemic forms. In the case of the US, which is the focus of this paper, it was hit the hardest and earliest by the pandemic because of weaknesses that were exacerbated and, in some cases, torn asunder as the three layers were bombarded. Each layer reveals independent dynamics, but as the US case demonstrates, the state’s ability to shore up extant fissures or exacerbate and create new ones has important effects on the experience of solidarity in everyday life.

## Disasters and Disintegration

Following a disaster, members of devastated communities tend to experience, individually or collectively, what is called *disaster syndrome* (Wallace 2003, pp. 149–163), in which survivors experience confusion, apathy, and passivity built on the

Perception that not only the person himself, his relatives, and his immediate property have been threatened or injured, but that practically the entire community is in ruins. The sight of a ruined community [in which the physical environment is] wrecked, is apparently often consciously or unconsciously interpreted as a *destruction of the whole world* (Wallace, 2003, p. 153, emphasis added).

Physical infrastructure, then, is social in both its external representation of the thing its members call community *and* in the facilitation of the everyday ritualized encounters and group life we come to take for granted as we *make* community. Disasters shred the moral anchors to which our personal identities are made and re-made and present severe barriers, in many cases, to finding old or new anchors. Without anchorage, we are unable to routinely and spectacularly plunge ourselves, to borrow a Durkheimian (1912 [1995]) phrase, in group life and reconstitute the very essence of society in ritualized encounters (Collins, 2004).

For instance, in Erikson’s (1976) study of a West Virginian hollow whose five tight-knit coal mining communities were destroyed by a massive flood, the hope of reconstituting what once was, was replaced by a pervasive collective trauma (Erikson, 1994). The cost of rebuilding was expensive and the damage extensive, but the flood also accelerated an already extant reality: the decline of intergenerational reproduction, which is a necessary ingredient for meso/micro solidarity. The initial confusion and apathy gave way to anomie and, instead of rebuilding their world, this anomic trauma became embedded in the community’s collective identity and many of the individual’s personal identities; a process or project not uncommon in most cases of social trauma (Alexander, 2004, p. 1). What we learn from these studies of disaster and trauma is that, one, disasters work on all levels and are very often local in their most enduring and direct effects. Additionally, the central problem of disasters and ensuing collective trauma is rooted in the very core of solidarity: how can the connective tissue tying the individual to their environment be rebuilt amidst an acute trauma that becomes a chronic, continuous problem? Reflecting on myriad local communities devastated by disasters, Erikson (1994, p. 242) concluded that our local milieu consists of “layers of trust” that surround humans,

radiating out in concentric circles like ripples in a pond. The experience of trauma, at its worst, can mean not only loss of confidence in the self but the loss of confidence in the scaffolding of family and community, in the structures of human government, in the logics by which humankind lives, and in the ways of nature itself (emphasis mine).

To put this in more general and contemporary sociological terms, these layers of trust are built up from repeated exchanges and interactions that generate affectual attachments to people, groups, and even abstract systems (Lawler et al., 2009) such that these social objects become an “extension of [our] own personality, an extension of [own] own flesh” (Erikson, 1976, p. 191). Though this is not a controversial metaphor, maintaining that this connective tissue is real, as Erikson (inspired by Durkheim) did, is controversial:

in much the same fashion as the cells of a body, [members] are dependent upon one another for definition, they do not have any real function or identity apart from the contribution they make to the whole organization and they suffer a form of death when separate from the larger tissue. . .It is the community that cushions pain, the community that provides a context for intimacy, the community, that represents morality and serves as the repository for old traditions (Erikson, 1978 p. 194, emphasis mine).

Tempting as it may be to write off the notion of a superorganism, the biological metaphor is not really as metaphoric as sociologists presume (Strassmann & Queller, 2010), especially on the local level where direct, reciprocal, recurring interactions take place (Fine, 2012). Humans are motivated to find and feel belonging, in part because of our evolved neuroarchitecture and heightened capacities for things like role-taking (Tomasello, 2020). Disasters, then, test the strength and durability of this connective tissue, but diseases are an especially problematic test.

For one thing, at least in the Western world, disease and plague are relatively rare, making people less prepared for the devastation. The notion of disease invites reflection on something old, primitive, Biblical, existential; meanings far removed from nuclear meltdowns. Diseases, unlike other natural disasters, are also unrelenting, at least until they run their course or meet resistance from human practice and invention. Complicating matters is the fact that they are invisible, biotic, organic threats, whose destructive path is not through razing physical infrastructure, but through *using* said infrastructure to create *disease chains*; both through mundane (grocery shopping), leisurely (tourism), and economic activities that constitute everyday life (McNeill, 1976). Finally, as disease courses through the social body, it infects the body politic, exposing weaknesses, exacerbating divisions, and testing the polity as the last protection against societal collapse.

Thus, COVID-19 presents an opportunity to think about how disease, as a special case of disaster, threatens mechanisms of solidarity on every level of social reality. It is tempting to begin at the macro-level, as broad brush strokes are so easy to discern. However, I prefer to start at the micro, as most of the anomic pain has been experienced in countless ways through the sudden loss of a wide array of moral anchors. The barriers to ritualized encounters due to disease, both unique and shared with other disasters, have shaken the foundations of the meso-level communal and associational groups that are the waters in which we routinely and spectacularly plunge and remake our self. Therefore, beginning with the everyday experience of disease makes more sense; however, it makes sense for a second reason: the effects of disease works its way “up” and not “down.” That is, threats to these rituals also threaten the always tenuous, but no less real, anchorage to the abstract, generalized, distant body politic.

Diseases present a particular challenge, as they neither strike the infrastructure nor the polity, at least not immediately. Like a zombie movie, they strike the very grounds of social and moral order: through ritualized interactions that simultaneously make the individual extraordinarily aware of the group’s external palpable, corporeal nature through the externalization and collectivization of emotion and attention *and* reinforce the social and moral order they have internalized through spectacular and mundane encounters in the past. Presumably, even highly generalized and abstracted rituals, like the celebration of an Independence or Memorial Day, serve to make the largest unit of political organization, the Nation-State, real and renew the attachment of the person’s identity to the increasingly distant collective identity. To carry the zombie analogy further, humans are the hosts of the threat, but unlike zombies, they are not often observably sick; especially asymptomatic carriers. All people are a potential risk. Many remedies for stopping a pandemic also naturally delimit interaction, as social distancing and mask-wearing serve as artificial barriers. The latter, in particular, runs counter to our evolved ape capacities, which direct us to pay especially close attention to face to determine threat, belongingness, identity, and so forth. In a sense, then, the polity and not the disease is responsible for the (metaphoric) destruction of physical infrastructure. Like a zombie movie, the most harrowing scenes are seeing recognizable landmarks where human activity should be abuzz, being empty and desolate, and the state’s efficacy in holding a diverse, geographically far-flung society together is challenged.

In short, as disease strikes at the heart of social solidarity, severing our ability to anchor ourselves to social objects from which we derive comfort, reward, and emotional/social/mental resources, the meso-level subsequently falls in disrepair as its life-blood - recurring daily interactions - are suspended. Consequently, pressure on the *idea* of what a society is, becomes ever-more tenuous as the very things that ward off *anomie* disappear (Abrutyn, 2019; Abrutyn & Mueller, 2016). These problems are exacerbated if society is already polarized and in conflict, no matter how dormant, along several different lines of stratification. Indeed, these lines have *always* become salient and legible amidst plagues (McNeill, 1976). Thus, while solidarity may be produced most directly and powerfully in local rituals that recreate the corporate units that externally embody their meaning and present tangible social and moral moorings to tether ourselves, it is large-scale institutions that stabilize and pattern these rituals. When these are threatened, people who are not in direct contact may experience similar feelings of anomie, putting real pressure on the polity to shore up the proverbial dam. Diseases are, unfortunately, hard to predict, as is the speed of their contagion, magnitude of morbidity, and duration of intensivity. States may react draconically; they may mismanage the crisis, thereby exacerbating a disaster; they may inequitably distribute resources in fighting the disease; and, they may pretend as though it did not exist, emulating Nero and his famous fiddling. Just as the barriers to ritualized encounters put pressure on the macro-level, the successes and, particularly, the failures of the polity, economy, science, and law return the favor, raising the stakes ever so high on the micro- and meso mechanisms of solidarity. For most readers, time and space were and perhaps still are objectively distorted as school, work, and home life are cast into chaos. As we slowly dig out of a disease-driven disaster, how we patch up, repair, and improve the invisible connective tissue, the webbing of group-affiliation that gives meaning and purpose to life presents its own challenges. It is these most tangible concerns, then, that give disease its greatest anti-social force; and, it is these concerns that I examine first in greater detail and specificity.

## The Phenomenology of Contamination

To make sense of a pandemic, I leverage a core conceptual tool that operates at the micro and meso levels: contamination. At the phenomenological level, plagues contaminate collective and individual actors—or, at the very least, threaten to pollute biologically and sociologically. This imagery derives in part from Durkheim’s (1995, p. 328ff) concern that the sacred must be protected from the profane, lest the former be polluted or contaminated; a point expanded in important ways by Goffman (1961, p. 23ff.; also 1963, 1967, p. 10ff) and his imagery surrounding the self and how it can be contaminated or spoiled. Essentially that which is owned, revealed, and enacted in collective life—including the self—must be set aside from that which is individual and driven by biological or utilitarian motivations. The underlying problem a pandemic poses, then, is two-fold. A disease obviously threatens biological infection, making every individual a potential threat of contamination. Social danger lurks everywhere. In addition, the efforts to mitigate spread, like social distancing, not only heighten awareness of the threat others pose, but also weaken or disintegrate social ties indefinitely. In Durkheimian terms, the threat of contamination produces anomic pain brought on by the sudden disruption in social life *and* in the sudden restriction of social reality. Consequently, collective actors struggle to remain viable, tangible things, as constituents choose to avoid or are mandated to avoid encounters; and when forced to enter into encounters, Goffman’s (1963) “rules” of stigma come into play, creating interpersonal problems for solidarity. At the meso-level, like a zombie movie, the physical and collective landscape appears to instantly unravel, leaving the usual environments for encounters and micro-level processes in the lurch, blocked, or totally disintegrated. To fully appreciate the dangers and horrors of the pandemic, let’s look at the most micro of micro-level processes: the sacralization of self.

### The Self as Sacred

The construction of a self and its location within a meaningful, purposeful, and supportive space begins in micro-processes related to interactions, exchange, and communication. Current neuroscience has identified the affective systems through which we are driven to seek out relationships that provide desired resources, the anger and fear we feel when those are threatened, and the panic and grief experienced when lost (Abrutyn & Lizardo, 2020). The point being that social relationships are built up from affect, which, in turn, acts as the underlying cohesive mechanism attaching individuals to each other and groups (Collins, 2004; Durkheim, 1912 [1995]), as well as to abstract systems and the imagined or generalized others associated with these systems (Lawler et al., 2009). Our self depends on these social or moral “anchors” for it to experience, again, the sense of belonging or connectedness. Hence, threats to or losses of those moral anchors generate panic, grief, as well as efforts to repair deteriorating relationships (Goffman, 1967), seek out new ones, or, potentially, adopt self-destructive or other-harm behavior. Our self, then, is not “ours,” it is forged and only meaningful within the context of the collective, and “on loan to [us] from society” (Goffman, 1967, p. 10). And, like any externalized, embodied representation of collective life, it is imbued with sacredness as collective effervescence spreads from ritual and saturates the self in “group-ness.” Efforts to protect the self, the other, and the underlying social bond demand vigilance, not merely in the practical sense of “caring” for a relationship, but also in a Durkheimian sense of purity and pollution (Douglas, 1966 [2002]). Diseases challenge the barriers we construct against contamination.

### The Spoiled Encounter

What happens when the principal method for both making the self sacred and for immersing oneself in the very thing that verifies one’s sacredness and generates transcendent meaning becomes threatening? What happens when daily life becomes marked by routine threats to pollute the biological *and* social self? Social life becomes difficult at best and, at worst, anomic. For Goffman (1961, pp. 23ff, 43–45), it was imperative and natural for humans to preserve and protect the self by cordoning off potentially polluting aspects of our environments. Ordinarily, humans use physical barriers—for example, relegating bodily functions to backstage, private space—in addition to discretion and tact when physical barriers are not available (Goffman, 1963). A pandemic constrains our active management of pollution, as the internal and external “territories of self [or] the boundary that the individual places between his being and the environment is invaded and the embodiments of self profaned” (Goffman 1961, p. 23). We are left with a difficult decision: self-isolate and risk being unable to enact self meaningfully or interact and risk contaminating the self and either becoming sick or, possibly, stigmatized for putting one’s own interests above public health.^1^ A final wrinkle: Like a good zombie movie, the source of contamination is not merely strangers or categories of “spoiled” actors (e.g., homeless), but rather our family and friends who are more likely to transmit the disease and, thereby, become both objects of concern and of potential pollution. Thus, it is not just extensive networks that crumble, but intensive ones, too, as the choice between potentially contaminating a loved one is thrown into sharp relief against being able to revivify that intimate, significant relationship.

This last piece is important in understanding another source of social and biological pollution; a source closely tied to the macro-level backdrop (most pronounced in the United States). While the mask is a reminder of the dangers posed by encounters, to *not* wear a mask has become a meaningful symbol of expressing animus toward one of the two “tribes” of Americans (Schneider, 2020); it is another example of how larger struggles over status, power, and meaning spill into mundane public encounters. On the one hand, mask wearers are willing to forgo social health to prevent biological contamination (as both a virtue signal of their commitment to the collective good and their personal health). On the other hand, mask-resisters see the restrictions and the threat of biological contamination as one more effort by a distant set of political, economic, and cultural forces (Hochschild, 2016) to threaten their individual choice (in the case of anti-“vaxxers”) and/or the social tissue of their communities (in the case of many conservative mask-resisters [Perry et al., 2020]). For the former, efforts to restrict biological contamination bring new dangers in routine encounters (like grocery shopping) designed to uphold the basic moral order, as the latter group has turned these spaces into performative sites of grievance, contest, and conflict. To be sure, these social dangers are compounded by the fact that those resisting Center for Disease Control (CDC) guidelines are also threatening to both catch and spread the disease to “essential workers,” usually drawn from marginalized communities and who sometimes lack the basic choice to wear or not wear, be exposed or avoid exposure (Krishnan et al., 2020). For the mask-resisters, the mask becomes a verifiable symbol of those agents complicit in the disintegration of their communities and their status and privilege (objectively true or not). Thus, unlike most disasters where uncontrollable forces affect us, the most painful and hidden aspect of disease is that biological and social contamination comes from *other people*. In sensationalized cases, it is the anti-masker; but in many cases, it is the fellow Church goer, an asymptomatic student, or a mildly symptomatic worker unaware of being infected.

A more subtle piece to this, one built on the generic processes of all diseases, but also on the social danger mask mandates have produced during COVID-19, is that contamination is not restricted to people, but also places. The disaster syndrome is not triggered by the loss of community as embodied in razed physical infrastructure, but in the phenomenological loss of space: the sudden fear of inaccessibility to places (and people) that are taken-for-granted extensions of our self, like the mailroom where we routinely check for correspondence, and the resulting deprivation of the sounds, sights, and smells intimately connected to the self. It is for these reasons, then, that some might extoll the virtues of digitally mediated interactions.

### The Digital Double Edge

Undoubtedly, the grief of isolation was temporarily abated by the ubiquitous use of digital communications technology that promised to resolve, at least, the practical problems of small talk, learning, and doing business. And, while online platforms have provided connections that would be lacking otherwise, there are four key downsides that may, in fact, exacerbate the negatives of interaction during a pandemic. First, and most tangibly, the collapse of the clear compartmentalization of kinship, economy, and education generated intense burdens on families, especially working moms whose fragile gains made in the public sphere are suddenly under siege. We have all become accustomed to a world in which we *do* family at certain times and places as apart from economic roles and school roles. Fear of spaces and government regulations instantaneously collapsed these boundaries.

A second problem stems from questions surrounding whether face-to-face encounters are superior to their digital cousins, an argument that rests on the suggestion that co-presence offers “ingredients” currently impossible to replicate online (Collins, 2004): the activation and heightening of our senses when palpably near other human bodies whose attention and affect are entrained and who bodies are synchronized. This assertion, admittedly, remains an open question. Strong evidence, though, points to severe limitations in digital interaction (Kalkhoff et al., 2020). Social interactions are both verbal and non-verbal, with both activating similar parts of the brain. Given that non-verbal is much older than verbal, it is at least equally important to conveying meaning about central aspects of a situation—for example, the actor’s own emotional disposition (Freedberg & Gallese, 2007). And, conveying meaning is imperative for encounters to achieve practical and phenomenological goals (Tomasello, 2020). Not surprisingly, some research has highlighted how digitally mediated interactions constrain what is visually available. For instance, children demonstrate deficits in learning from screens (Krcmar, 2010), which is tied to differences in neural processing (for a review, see Dickerson et al., 2017). It is perhaps for these reasons, and of course, cultural ones as well, that for some people digitally mediated forms of interaction are felt as hollow and difficult to embrace (Tufekci & Brashears, 2014), which undoubtedly affects how committed an actor is to the success of a given encounter and restricts emotional entrainment.

For youth, these first two problems are exacerbated by a third: their once stable (in-person) social worlds shift to a cocktail of online learning and social media connectivity. Gone are the rituals of high school that, for better or worse, characterize what youth and adults define as “normal” childhood. The efficacy of social media in providing the developmental and support needs of youth remains dubious (Holtzman et al., 2017), while social isolation during online or hybrid learning runs counter to what appears to make youth most healthy (Orben et al., 2020).

The fourth problem comes from a surprising body of empirical research; one that points to the virtues of digitally mediated interaction. It has been shown, for instance, that these platforms can facilitate the emergence of *digital affect cultures*, or artificial environments that facilitate and heighten the flow of affect from user to user by generating emotional resonance and alignment, can build some form of community (Döveling et al., 2018). While this research does push back against the idea that online encounters are *always* unsatisfying, it does raise another issue: when digital mediation is successful in protecting against biological contamination while mitigating the structure of social health, does it prevent the spread of social contamination? It is clear that not all connectedness is a good thing (Abrutyn & Mueller, 2016), as encounters and ritualized interaction, especially with people we trust and care about, facilitates the diffusion of feelings, thoughts, and actions. Emotions are especially “contagious” or easily diffused through encounters as humans disclose their struggles, both explicitly and implicitly. In short, better connectedness amidst a pandemic means more efficacious exposure to others’ suffering and experiences during the pandemic, which may contribute to the diffusion of a shared sense of anomie. Thus, even if digitally mediated encounters do provide us with ritual and assembly for plunging our self into, it also allows for rapid diffusion of a discourse of danger, risk, contamination, and real or false beliefs about the immediate or long-term future.

### From Micro to Macro

Against this backdrop of a sacred self yearning to ward off creeping anomic pain as ritualized interactions, collective assemblies, and regions of everyday life become threats of contamination, the macro-world around us distributes choices, experiences, and contamination unevenly. Put differently, while the local world we inhabit is the true source of solidarity, these smaller units of life—encounters, groups, organizations—are nested within a large structure and culture that directly and indirectly permeates the construction of solidarity within them and between those units and others. In the United States, for instance, the horrors of the pandemic in the earliest months cannot be fully grasped without considering political polarization. For many “Blue State” denizens, the polity’s (and, more specifically, then-president Trump’s) inefficacious, incompetent response only served to destabilize social reality even more. Directly, by calling into question whether the state could protect its citizens, and indirectly as “Red State” individuals were both emboldened by mixed messages from the president, the messaging of the conservative media, and by their own fear of their shrinking social webs getting any smaller and, consequently, turned the public sphere into sites of direct conflict. Structurally, pre-existing cleavages (e.g., racial divides) were exploded by both the inequitable burden of risk spread across racial, ethnic, gendered, and class lines, extant inequities in health care access, the tendency to live in denser, multi-generational communities, as well as by the more general stress and anxiety folks felt in the face of biological and social contamination; these forces made civil society a tinder box. As with previous polities threatened by disease, its weaknesses were exposed alongside economic and civic weaknesses, which is why the US experienced the pandemic so much more intensely (at first?) than, say, Canada or Germany. Thus, it is imperative that we examine the macro-level dynamics of solidarity in light of those described above.

## Institutions and Social Solidarity

Before examining the specific context of the US, however, it is worth taking a step back and thinking about what institutional spheres are and why polity matters so much to solidarity in the face of disaster and disease. Although *institution* is notably vague, I define institutions as the macro-level spheres or social orders that are the fundamental bedrock of all human societies. Universal spheres, such as kinship, polity, religion, law, and economy, organize beliefs and practices for significant portions of a given population, as do more recent spheres like science or medicine.^2^ Viewed this way, institutions are the bedrock of any society’s ability to integrate far-flung, heterogeneous populations. They do so, at the most basic level, by subjecting batches of people to generalized structural and cultural conditions: role/status relations like doctor–patient, mother–child, or teacher–student act as everyday vehicles for imposing generic rules and regulations (while allowing for flexibility, local variation, and personal idiosyncrasy) on any and all who inhabit those positions. Finally, by embedding encounters, groups, and organizations in a common cultural space, they integrate people through mundane and occasionally spectacular rituals designed to connect individuals with the distant institutional center.

Rather than see institutions as reifications, then, I treat them as physically, temporally, socially, and symbolically real in the effects they have on our experience of daily life. Thus, as polity or science becomes increasingly autonomous (i.e., its physical, temporal, social, and symbolic spaces grow structurally/culturally discrete vis-à-vis kinship or religion), it acquires cognitive sovereignty over a set of processes, practices, and problems. We feel, see, touch institutions in both the arrangement of space in their domination and authority over certain problems, practices, beliefs, and so forth. By taking one of the generalized roles (e.g., patient, client, citizen), we take for granted that their counterparts will fill in our blind spots; they assume a host of risks for us, presumably, for our benefit.

Central to my analysis is the polity, or the institutional sphere responsible for collective, binding decision-making, producing and distributing power, and mobilizing resources to realize whatever goals its elite set. Typically, polities are highly pragmatic institutional spheres, even when legitimated religiously, as they are the primary response to exigencies of all types. In centralizing risk management, polities deal with public works, defense, third-party adjudication, and so on (Johnson & Earle, 2000). In the past, polities used whatever mechanisms were available, though without Germ Theory, its responses were limited. Hence, the importance of science in modern efforts to deal with disasters, especially disease (Beck, 2008). Consequently, micro-processes of solidarity must be understood as filtered through and reciprocally acting on the existing institutional complex or arrangement of a given society. Theoretically, a strong, positive response that appears to be protective will provide a barrier against the threats to our everyday experiences, whereas a weak or incompetent response will not only expose the lack of a safety net, thereby increasing the anomic response, but also will have real consequences for how risk is distributed. The State’s inequitable response under ordinary conditions, particularly those historically burdened by the uneven distribution of resources, exacerbates these conditions and further widens already disintegrative fissures. In what follows, I examine the more general suppositions surrounding institutions, solidarity, and disease and then some key details that underscore why the US has been especially vulnerable to the pandemic, particularly leading up to the 2020 election.

### Institutional Risk

To some extent, we live in what Beck (1992, 2008) termed a “risk society,” or a world in which the identifying, processing, and triaging of—and, I might add inoculation against—risk has been increasingly centralized into the structure and culture of the polity and its reliance on science. Centralization, as it always has been, is both a source of risk—especially in highly bureaucratized states that often ruthlessly determine what a hazard is and how to calculate reasonable loss—and a source of solution given its monopoly over implementing policies meant to contain or reduce risk. Besides the shadow of political expediency that shapes risk management, the polity’s principal weakness—again, exacerbated in modernity—is the fact that they are arenas in which status groups and class interests, usually filtered through party machinations, clash. Put differently, States are typically rational and thus operate on expedient decision-making (Scott, 1998), but this expediency is greatly shaped by political agendas of parties in control and those striving to take control. Because political parties vary in their composition, control over the state can exacerbate (or dampen) existing patterns of inequality that lead to the off-lining of risk to some disadvantaged regions, populations, and, in the world-system, nations.

Typically speaking, polities rarely “win” against diseases, though they may outlive them. Sometimes this is because polities respond inefficaciously, but the principal reason polities struggle is that diseases are “irrational” and rooted in their contaminative capacity. For instance, the first States, built on alluvial flood plains *invited* their own destruction as canals and irrigation also gave rise to a new barbarian and threat to “civilization”: a water-borne disease, schistosomiasis. Polities continuously stumbled and fell whenever schistosomiasis spread through the farmlands, causing debilitating lethargy and sudden, insurmountable shortages in food (McNeill, 1976). Their stealthy, illegibility makes diseases resistant to political solutions, in many cases, and, therefore, elusive to risk management. Not surprisingly, in ancient states, the invisibility, lack of control, and sudden devastation conditioned a discourse and set of techniques fashioned around the idea of danger. Dangers are built on invisible, blameless calamities and exigencies (Luhmann, 2005), and require an older, morally “thick” language that contrasts with the technocratic nature of risk (Douglas, 1990). Thus, while Beck argues that problems in postindustrial, postmodern societies are identified, processed, and triaged through risk discourse, COVID-19 has shattered the idea that modern humans are superior to their premodern ancestors. The forensics of disease are refracted through a discourse of danger, not risk; the latter proves too sterile, too antiseptic to manage the contaminative edge that is experienced in daily life. A side effect of the danger discourse is the moral nature of blame, a factor that adds to the threat of social contamination and the construction of diffuse beliefs about who is to blame as well as what classes of people pose the most danger. Thus, while the institutional apparatus appears to be failing, people turn to either the State or extra-State actors to erect new and strengthen old barriers between those deemed “normal” and those who are dangerous.

To be sure, these inherent institutional weaknesses were exacerbated by, say, the US government’s response. The political calculus of Trump made for a volatile mix with the conservative position in the culture war against any intrusion by the State in personal, local, or state affairs. Unfortunately, the polity’s reliance on the scientific institution for risk management techniques further exposed the weaknesses of institutional solutions, as inherent weaknesses in science combined with the Trump administration’s weak response to reduce confidence and trust in scientific techniques.

Where polities deal in expediency, science deals in probabilities, knowledge accumulation, and the unattainable and intangible goal of truth. Its fruits are enjoyed in material pleasures such as air conditioning, air travel, and the internet. But, science has never been effective at dealing with the three ontological problems identified by Geertz (1966): uncertainty, suffering, and evil, because, in its ideal typical form, science is disinterested and lacks an affectual connection. It may explain why a person’s heart gives out, but it offers no comfort or solace. Indeed, rather than answer ontological dilemmas, science has sometimes exacerbated them—for example, Nagasaki/Hiroshima. However, the blending of science and polity in risk societies has had a decent track record, at least in terms of public health, since the 19^th^ century, which is why a pandemic is so interesting; and horrifying. Most notably, the State’s inability to contain the disease, much like the ineffectual nature of States in zombie movies, and science’s inability to immediately diagnose and treat the disease (as unfair an expectation as that may be), not only hastened biological contamination but also presented a new social danger. In part, science’s “failure” was due to its own cognitive and emotional limitations built upon the blind spots inherent in any community of knowledge producers (Molteni, 2021). But, science was also unfairly put to the test in a 24-hour world driven by instantaneous demands for results. Either way, COVID-19 raised significant questions about how safe the State and science make us *or can make us*.

The final piece that helps explain risk management and its inability to contain dangers stems from the economic system that undergirds all human activities, including politics and science, is capitalism. Western states rely on consumption, and consumption requires employment and a lack of interruption in economic activities. The US was stuck between its commitment to the collective and to sustaining the economic sphere, exposing further weaknesses in the institutional abilities to avoid danger and maintain social solidarity. In short, history repeats itself: an invisible “barbarian” has done what social movements of all sorts have failed to do: expose the tenuousness and exploitative nature of the institutional apparatus that presumably stabilizes and encourages solidarity. Apparatus that is further weakened by existing political, economic, and cultural fissures that dot any given nation’s landscape.

### Risk Refracted Through Personal and Impersonal Societies

Solidarity, then, is endangered by the polity’s ability to keep the fissures from cracking, but a polarized polity (Beck 2008, p. !2), like the US has its hands tied behind its back as it navigates the containment of contamination. To oversimplify for the sake of argument, the US is largely divided into two tribes. Risk and danger are perceived differently by these two different parts of the US, in part because of the micro/meso mechanisms by which the self is sacralized. The conservative image of community envisions the moral center as palpable, tangible, and directly available. Here, actors commune directly with their moral center, together in collective assemblies composed of mundane interactions at the post office and spectacular Sunday rituals in a (mega) church (Wuthnow, 2018, pp. 30–32). Their direct, local experiences have become defined as *American* and thus threats to *it*, to the ability to *do community*, are threats to both the moral center and each member’s personal identity. In a penetrating study of the federal government’s role in protecting citizens against environmental dangers, these feelings were clearly expressed over and over again. “It was hard enough to trust people close at hand, and very hard to trust those far away; to locally rooted people, Washington D.C. felt very far away [resulting in a threat of] frightening loss. . .of their cultural home, their place in the world, and their honor” (Hochschild, 2016, p. 54). For members of small towns, the horrors of the plague are less rooted in the threats to the individual self, but rather in solidarity-producing moral anchor. This notion of risk is buttressed by the local assemblage of institutional space: science is replaced by a particularistic, local religio-kin type system (that includes members *in* the community as fictive kin through extensive moral ties) fused with community politics, which, in turn provide a protective barrier against the distant, a/immoral national polity and global economy (Whitehead & Perry, 2020). Consequently, the danger discourse shifts the blame to threats to local autonomy (the “nanny state”), liberal transgressions against Christian values, and modernity’s threat to the traditional family.

For the other “half” of America—if that is not too simplistic a label—the pandemic casts the cohesive nature of the US in doubt, too. In part, this is due to the specific time and place: the Trump administration was not viewed as legitimate by many and its eponymous leader was viewed as a real threat to what this segment deems *American*. Like Nixon and Watergate (Alexander, 1988), Trump is perceived as polluting the political center on a spiritual and practical level. The chaotic response—for example, throwing away the Obama pandemic “playbook” (Knight, 2020)—undermined the polity’s capacity to practically and symbolically contain the contamination and reflected the belief that the state had become a patrimonial tool for the pleasure of the administration; a situation that was not simply offensive on a cognitive level, but a moral level for many in this US tribe. Like their small-town counterparts, these communities reached the same conclusions, but for different reasons: the federal government’s claim to cognitive sovereignty was doing more harm than good. The two tribes saw the solutions as different, with the conservative response being more local autonomy (Perry et al., 2020. and their counterparts being in favor of a stronger, more competent State response (Haffajee & Mello, 2020). This divide easily mapped onto the everyday experience of danger that Blue State denizens faced or feared they would face, as dense urban spaces not only facilitated biological contamination but increased the opportunities to encounter social dangers posed by mask-resisters, including as well as anti-“vaxxers” who tend to disproportionately live in urban areas (Olive et al., 2018).

In short, the weaknesses of the American institutional apparatus made salient the cleavages, inequities, and polarities that, in times of calm, are often washed over by economic life. Besides the ideological divide highlighted above, economic and racial inequalities have also predictably become pressure points that have both exposed the weaknesses of the polity and made it weaker at the same time. Though it would be naïve to minimize Black American’s grievances or the working class’s precarious position, it is plausible to wonder whether these divides would have bubbled to the top so ferociously had the polity handled the pandemic more effectively. The phenomenological terror of social danger, biological risk, and the inability to reproduce self and the social/moral order leaves folks feeling acute anomie. And, it is this macro-level layer of anomie combined with the social and biological threats tearing at the fabric of everyday life that has driven protests, civil unrest (among “both” Americas for different reasons), and, at best, made a hopeful future cloudy; and at worst, impossible.

## Final Thoughts

In spite of his horror and pessimism, Durkheim’s sociology expressed optimism in humanity. Disruptions, though potentially fatal for society, also brought opportunities to reshape how things are. After the Spanish Influenza killed 50 million worldwide (650,000 of which were Americans) and infected scores more, the world went back to “normal.” The “roaring 20s” in the United States was filled with gatherings of many people; baseball stadiums were soon filled; and, the handshake remained to this day, the principal greeting in polite and depersonalized society. Perhaps, then, 2020 (and, probably 2021) are blips on an otherwise steady march toward greater empathy and equity? The argument against this optimism, however, points to the historical context of the Spanish Influenza: the 1920s followed on the heels of World War I, in which a significant number of young men were killed alongside the tendency for the flu to kill younger people (relative to COVID-19s mortality rates). Likewise, the US, today, is very different from the 1920s, when Jim Crow laws reduced the need for White communities to find ways to generate solidarity with communities of color, especially Black communities. Perhaps repairing the US and building solidarity seems more difficult today than in the early 20th century.

Setting aside these longer-term questions, it is also reasonable to ask two related questions: how long will COVID-19s effects linger? The vaccines seem effective against many variants, but the resistance to vaccines throughout the West and the inequitable access to them throughout the world has complicated the slowdown of COVID-19. The discourse of danger also threatens to crystallize mask-wearing and social distancing as signs of belongingness to one political tribe vis-à-vis another. What might no longer be necessary for public health, may remain markers of moral distinction. The second question is premised on the assumption that this is not the last pandemic contemporary humans will experience. Do political and scientific institutions learn from their successes and, more importantly, failures, or are diseases so unpredictable that we are doomed to repeat our mistakes? So long as the US remains polarized, it is difficult to imagine the US adapting, though much of the rest of the world may be better prepared.

As we move down to the meso and micro-levels of social reality, we see more immediate changes possible. The US in the 1920s was already accustomed to belonging to voluntary associations. Perhaps the pandemic has laid bare just how much we miss these types of organizational attachments. The reliance on digitally mediated social space continues to prove, neurobiologically and sociologically, unsatisfying for most (Kalkhoff et al., 2020; Tufekci & Brashears, 2014), and thus, a trend toward more “joining” may arise if and when a sense of safeness replaces that of danger. It is also possible that Americans are motivated to re-make the idea of joining, and hybridize modern life with traditional collective life. Youth have been leaving dense urban spaces for some time, and it may be the case that the suburbs are reimagined with multi-generational family life emerging in planned communities that thrive on distance employment.

Perhaps more interesting is what the post-pandemic collective American identity will look like, especially among young Americans who have come of age post-9/11 and have lived through some highly traumatizing periods of time. Though they fought in no wars like their grandparents or great-grandparents, they have endured myriad economic downturns, highly visible displays of racialized violence, and an impending sense of hopelessness as climate change has, thus far, remained beyond their control. Collective and cultural trauma is all about identity (Alexander, 2004; Erikson, 1994): groups can incorporate the experience and interpretation of trauma into their own sense of self, elevating it to one of, if not the, defining attribute of who they are. The self can only sustain being defiled and contaminated for so long before defensive measures are put in place. What COVID-19 does to these cohorts, both directly in the experience of masks and social distancing, and indirectly in the form of economic vulnerability, racial injustice, and civil unrest remains to be seen.

Tempting as it may be to end on a fatalistic note, it is imperative to contextualize the now and push back against the allure of “golden ageism.” Human societies have faced severe pressures in the form of wars, diseases, natural disasters, and so forth several times over. The stakes are higher now only because the size and scale of failure are exponentially greater. But, telling the future is not social science’s strength precisely because human ingenuity has been our greatest adaptive characteristic. Just as collapse is commonplace in history, so too are resilience, reconfiguration, and revival.

## Notes

1. Of course, these choices are unevenly distributed, which add to a history of imposed profanation of self for many marginalized individuals and communities.

2. For an extensive review of institutions presented herein, see Abrutyn and Turner (2021).
